# Association of Natural Resistance–Associated Macrophage Protein 1 Gene With ND Antibody Titer and Leukogram Parameters in Local Chickens

**DOI:** 10.1155/vmi/5243434

**Published:** 2026-05-25

**Authors:** Ismoyowati Ismoyowati, Janet Mamutse, Setya Agus Santosa

**Affiliations:** ^1^ Department of Poultry Production, Faculty of Animal Science, Jenderal Soedirman University, Purwokerto, Central Java, Indonesia, unsoed.ac.id; ^2^ Department of Animal Sciences, Faculty of AgriSciences, Stellenbosch University, Private Bag X1, Stellenbosch, 7600, South Africa, sun.ac.za; ^3^ Department of Agriculture and Animal Health, College of Agriculture and Environmental Sciences, UNISA Science Campus, Florida, Johannesburg, South Africa; ^4^ Department of Animal Breeding, Faculty of Animal Science, Jenderal Soedirman University, Purwokerto Central Java, Indonesia, unsoed.ac.id

**Keywords:** chickens, immunogenetics, Newcastle disease, NRAMP1 gene, SNPs

## Abstract

Newcastle disease (ND) is a major infectious disease impacting chicken productivity. The natural resistance–associated macrophage protein (NRAMP1) gene plays a critical role in the innate immune response. This study aimed to evaluate the association between NRAMP1 gene polymorphisms, ND antibody titers, and leukogram parameters in three local Indonesian chickens: Kampung, Red *Kedu*, and their crossbreds. Each group consisted of 50 four‐week‐old chickens. Blood samples for ND antibody titers (via hemagglutination inhibition test) and total and differential leukocyte counts were collected 21 days postvaccination. DNA for genotyping was extracted at 8 weeks of age. Genotyping was conducted through sequencing. Data were analyzed using ANOVA, with post hoc comparisons by Duncan’s multiple range test. Genetic parameters such as allele/genotype frequencies, heterozygosity, and genetic distances were also determined. Results revealed three genotypes at the NRAMP1 locus. The CC genotype predominated in Red *Kedu* (40%) and crossbred chickens (41.5%). A strong correlation was observed between the NRAMP1 genotype and ND antibody titer (*r* = 0.744, *p* < 0.01). Additionally, genotypes were significantly associated with leukogram parameters, namely, heterophils (*r* = −0.858), lymphocytes (*r* = 0.855), and H/L ratio (*r* = −0.862). Chickens with the TT genotype showed higher ND antibody titers, increased lymphocyte percentages, and lower H/L ratios, indicating better immune response and lower stress. Allele frequency analysis showed a slightly higher prevalence of the C allele (0.515) over *T* (0.485). The *T* allele had positive additive effects on ND antibody titers and lymphocyte percentages. Breeding value estimates suggested that the TT genotype possesses superior genetic potential for immune‐related traits. High genetic variance for ND titers also highlighted significant biodiversity, underscoring the value of NRAMP1 polymorphism in breeding strategies for disease resistance. Therefore, this study suggests that the NRAMP1 gene has potential as a biomarker for ND resistance and could be considered for use in breeding programs of the Indonesian local chickens. The study recommended further studies to investigate chickens that are challenged with a virulent ND strain to validate findings from this research. Furthermore, future studies should consider sequencing the complete fragment of the NRAMP1 gene.

## 1. Introduction

Various poultry breeding programs have been developed to improve productivity and disease resistance. Recently, resilience in poultry has gained prominence as an alternative strategy to improve productivity while reducing production costs [1]. Indigenous chickens are particularly important in rural economy and backyard farming systems across many countries [2]. Local chickens play a crucial role in household food security in rural areas of developing countries [3]. In Indonesia, the population of local chickens reached approximately 314,101,000 in 2022, accounting for approximately 8.16% of the total chicken population (Directorate General of Livestock and Animal Health Services, 2022). Among the various indigenous breeds, Kampung chickens (*Ayam Kampung*) and *Kedu* chickens (*Ayam Kedu*) are known for their high potential to produce both meat and eggs. *Ayam Kampung* is a dual‐purpose local chicken with the purpose to produce both meat and eggs [4]. In contrast, *Ayam Kedu* is a local breed originating from the *Kedu* region, primarily recognized for its relatively high egg production, although its population has recently declined [5]. The phenotypic characteristics of *Ayam Kedu* vary by strain. For instance, the Black *Kedu* strain is characterized by black plumage covering the head, neck, body, shanks, and tail, along with a reddish‐black single comb. The Red *Kedu* strain similarly exhibits black coloration across most of the body but has white skin, yellowish‐black shanks, and a red or dark‐red single comb [6]. Crossbreeding Red *Kedu* roosters with *Ayam Kampung* hens has led to the development of local chicken refered to as UNSOED chickens.These UNSOED chickens exhibit a heterosis effect of 7.8% in egg production [7].

Efforts to improve resistance to infectious diseases, such as Newcastle disease (ND), are an important goal in poultry breeding programs. The heritability of the disease resistance to ND has not yet been estimated due to the difficulties in measuring the phenotypes and in distinguishing between high and low levels of resistance. Marker‐assisted selection (MAS) is one of the molecular genetics approaches that is ideal for selecting chickens with resistance traits against ND and higher immunity levels based on the genes associated with immunity in local chickens [8].

Natural resistance–associated macrophage protein (NRAMP1) is a crucial protein involved in iron ion transport, particularly in the removal of iron from macrophages [9]. This function is vital in the innate immune response, as iron availability can influence pathogen survival. The NRAMP1 is ubiquitously expressed across various cell types and is typically upregulated in response to pathogen infection in various livestock species [10, 11]. The NRAMP1 gene has been identified as a candidate gene associated with disease resistance in a range of domesticated animals [12] and is specifically linked to immune traits in chickens [13]. In chickens, a homolog of the NRAMP1 gene has been mapped on chromosome 7, where it is located within the promoter region. The gene structure comprises 15 exons and 14 introns, with a total nucleotide length of 5760 bp [14]. Advances in molecular technology have significantly contributed to new insights into the genetic improvement of disease resistance in poultry, particularly through the identification and application of disease resistance–related genes [15].

Single‐nucleotide polymorphisms (SNPs) represent the most common form of genetic variation in the genome. In coding genomic regions, SNPs can be classified into two types, i.e., synonymous SNPs, which do not alter the amino acid, and nonsynonymous SNPs, which do alter the amino acid sequence. Nonsynonymous SNPs are of particular interest due to their potential impact on gene expression and protein function, whereas synonymous SNPs are less likely to affect gene expression. Nonetheless, both types serve as valuable markers for mapping studies [16]. Previous studies have demonstrated that the NRAMP1 gene influences the antibody titers against ND in SenSi‐1 Agrinak chickens, showing a significant correlation with the TC genotype [17]. Therefore, the present study aims to identify SNPs within the NRAMP1 gene in *Ayam Kampung* and *Ayam Kedu* strains and to evaluate their association with ND antibody titers and leukogram parameters.

## 2. Methodology

### 2.1. Animals and Sample Collection

This research was conducted at the experimental farm and the biotechnology laboratory of the university. This study used three chicken breeds including Indonesian local chickens, *Kedu* chickens, and cross between *Kedu* and Indonesian local chickens. Each group consisted of 50 birds that were 4 weeks old. The study was approved by the Ethics Committee of the Faculty of Veterinary Medicine, Gadjah Mada University (no. 007/EC‐FKH/Eks.2024).

The chickens were reared from 4 to 12 weeks of age. Wing bands were used for individual identification. The chickens were housed in litter‐floor pens measuring 3 × 4 m (12 m^2^), maintained at a temperature range of 26°C–29°C, a relative humidity of 65%–70%, with 12 h of light provided daily. Feed was provided in measured amounts according to age, 30 g/bird/day at 4 weeks of age and increasing to 70 g/bird/day at 12 weeks of age. The feed comprised ground corn (45%), rice bran (33%), and concentrate (22%), with a nutritional composition of 2900 kcal/kg metabolizable energy, 16.10% crude protein, 5.61% crude fiber, 5.23% crude fat, 2.52% calcium (Ca), and 0.86% phosphorus (P). Water was provided ad libitum. At 5 weeks, all chickens were vaccinated against ND using Medivac ND Emulsion (produced by Medioan Farma, Indonesia).

Blood samples for ND antibody titer, total leukocyte count, and leukocyte differential counts were collected 21 days postvaccination from all chickens. Blood for DNA extraction was collected at 8 weeks of age.

### 2.2. Leukocyte Profiling and ND Antibody Titer Measurement

The Turk’s solution and Giemsa stain were used for leukocyte and differential leukocyte analysis. The ND antibody titers were determined using the hemagglutination inhibition (HI) test on serum samples. Total leukocyte counts were performed using a Neubauer improved hemocytometer [18]. In this study, we did not consider investigating antibody titers in nonvaccinated chickens (control group) as antibodies are undetectable unless the chickens have previously been exposed to ND or AI infections.

### 2.3. Identification of NRAMP1 Gene Polymorphism

DNA was isolated using a commercial DNA isolation kit, followed by PCR, which was also done using a commercial PCR kit. Subsequently, the PCR products were genotyped using the single strand conformation polymorphism (SSCP) method, and the PCR–SSCP products were visualized using electrophoresis on acrylamide gel. Furthermore, the PCR products were sequenced and visualized as electropherograms with nucleotide bases identified by color: green for adenine (A), black for guanine (G), blue for cytosine (C), and red for thymine (T) [19]. Sequence analysis was conducted using MEGA 6 software [20] and BioEdit software [21] by aligning the sequences with the NRAMP1 gene reference (GenBank accession number AY072001.1.) to identify SNPs.

### 2.4. Data Analysis

Data was analyzed using the analysis of variance (ANOVA), with chicken strain (local, *Kedu* and crossbred) as the independent factor to determine its effect on ND antibody titers, leukocyte counts, and differential leukocyte counts. Post hoc analysis was performed using Duncan’s multiple range test (DMRT). Allele frequency, genotype frequency, heterozygosity, and genetic distance were calculated using the following formulas:

Allele frequency (1):

(1) xi=2nii+∑nij2N,

where

 
*xi* = frequency of the *i*th allele
 
*nii* = number of individuals with genotype *ii*
 
*nij* = number of individuals with genotype *ij*
 
*N* = total number of individuals in the sample

Genotype frequency (2):

(2) xij=niiNx100%,

where

 
*xii* = frequency of homozygous genotype (*ii*)
 
*xij* = frequency of heterozygous genotype (*ij*)
 
*nii* = number of individuals with genotype *ii*
 
*nij* = number of individuals with genotype *ij*
 
*N* = total number of individuals in the sample

The gene effect [22, 23] (3):

(3) p+q22221=p+pq+q=.

Breeding value:

(4) A11212A=a=qa,A1212A=a+a=q−pa,A22222A=a=−pa.

Dominance deviation (D) = {Genotypic value (G) − Breeding value (A)}, or (*D* = *G* − A) (4):

Additive variance (VA):

(5) =p222qa+22pqq−pa+q22−2pa=22pqa.

Dominance variance (VD):

(6) =p22−22dq+22pq2pqd+q22−22dp=4222pqd.

Genetic variance (VG):

(7) =24222pqa+pqd.

Heterozygosity (5):

(8) He=1∑i=1npi2,

where He = heterozygosity, *n* = number of alleles, *i* = allele, Pi = frequency of allele I,

Genetic distance (6):

(9) D=−lnGxy/GxGy,Gx=∑pi2,Gy=∑qi2,Gxy=∑piqi.

## 3. Results and Discussion

The NRAMP1 gene works synergistically with macrophage cells to fight pathogens. The expression of the NRAMP1 gene in macrophage cells facilitates the engulfment and killing of pathogens or bacteria that have penetrated tissues. Several studies have shown that NRAMP1 is among the genes that influence disease resistance in chickens [11]. Muhsinin et al. [24] also reported that the NRAMP1 gene is associated with immune traits in Indonesian local chickens.

Table 1 shows the mean values of the ND antibody titer and leukogram parameters in the three chicken strains investigated. Furthermore, the findings of NRAMP1 genotyping in the three chicken strains showed the presence of polymorphism in the gene. The genotypes were confirmed by the electropherograms in Figure 1. The identified genotype and allele frequencies in the three local chicken populations are presented in Table 2.

**TABLE 1:** Mean ND antibody titer and leukogram parameters in local, *Kedu*, and crossbred chicken strains.

Chicken strains	ND antibody titer	Total leukocyte count (cells/uL)	Heterophils (%)	Eosinophils (%)	Lymphocytes (%)	Monocytes (%)	H/L
Local chickens	352.653	9551.020	30.367	2.489	60.204	6.939	0.529
*Kedu* chickens	254.439	9886.585	32.171	3.219	58.390	6.219	0.599
Crossbred chickens	201.366	9919.512	31.268	3.049	59.341	6.341	0.565
SEM	31.698	149.198	0.809	0.139	0.783	0.209	0.022

FIGURE 1Electropherogram of the NRAMP‐1 gene showing genotypes CC (a), TT (b), and CT (c) at 163 bp.

**Figure:**
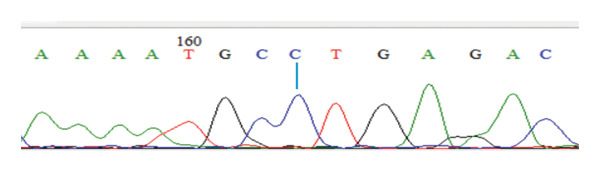
(a)

**Figure:**
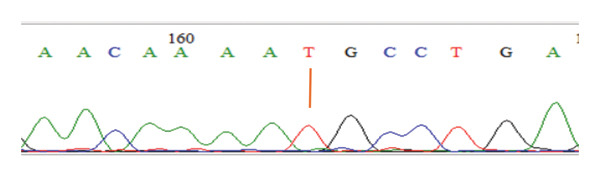
(b)

**Figure:**
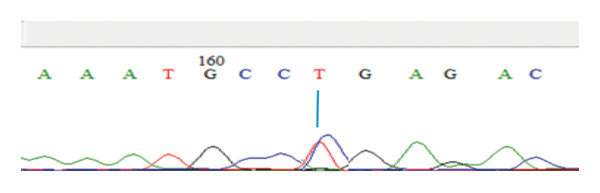
(c)

**TABLE 2:** Genotype and allele frequencies based on the NRAMP‐1 gene in three chicken strains.

Chicken strain	*N*	Genotype frequency			Allele frequency	
		CC	CT	TT	C	T
Local chickens	49	0.354	0.250	0.396	0.479	0.521
*Kedu* chickens	40	0.400	0.300	0.300	0.550	0.450
Crossbred chickens	41	0.415	0.195	0.390	0.512	0.488

**FIGURE 2:**
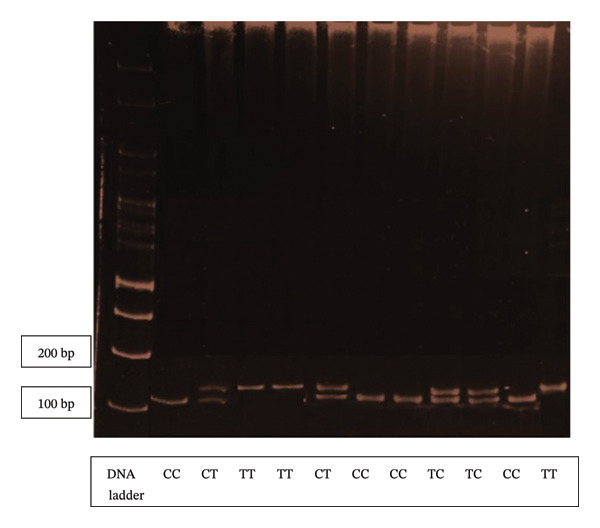
SSCP–PCR product of the NRAMP1 gene in local Indonesian chickens.

The local chickens showed the highest ND antibody titers while the leukogram parameters varied across populations. The findings of SSCP–PCR genotyping of the NRAMP1 gene identified three genotypes, TT, CC, and CT (Figure 2). The homozygous CC genotype had the highest frequency in *Kedu* and crossbred chickens (40% and 41.5%, respectively), while it was the lowest in local chickens (39.6%). On the other hand, crossbred chickens had the smallest frequency of the heterozygous genotype (19.5%), compared with the local and *Kedu* populations, which had frequencies of 25% and 30%, respectively. Furthermore, the frequency of the *T* allele was lower than that of the C allele in all three chicken strains although the difference is quite small (0.485 vs 0.515). These findings align with previous studies that reported the CC genotype of the NRAMP1 gene having a higher frequency and the TT genotype being low in local Indonesian chickens [11, 17, 24].

The local chickens showed the highest ND antibody titers while the leukogram parameters varied across populations. The findings of genotyping polymorphism identification in the NRAMP1 gene showed that the homozygous CC genotype had the highest frequency in *Kedu* and crossbred chickens (40% and 41.5%, respectively), while it was the lowest in local chickens (39.6%). On the other hand, crossbred chickens had the smallest frequency of the heterozygous genotype (19.5%), compared with the local and *Kedu* populations, which had frequencies of 25% and 30%, respectively. Furthermore, the frequency of the *T* allele was lower than that of the C allele in all three chicken strains although the difference is quite small (0.485 vs 0.515). These findings align with previous studies that reported the CC genotype of the NRAMP1 gene having a higher frequency and the TT genotype being low in local Indonesian chickens [11, 17, 24, 25].

The correlation between NRAMP1 genotypes (CC, TT, and CT) and the ND antibody titer had a correlation coefficient of 0.744 with *p* < 0.01, indicating that the NRAMP1 gene significantly affects ND resistance. The correlation analysis showed that the NRAMP1 genotype were also strongly associated with the parameters of heterophils, lymphocytes, and the H/L ratio. The correlation coefficients for the genotype with the heterophil fraction were −0.858, with the lymphocyte fraction were 0.855, and with the H/L ration were −0.862. Therefore, NRAMP holds promising potential as a genetic marker for selecting chickens with high resistance to ND in breeding programs.

Furthermore, chickens with the TT genotype had the potential for higher synthesis of ND antibody titers, a higher percentage of lymphocytes, and the lowest stress levels, as indicated by the lowest H/L ratio compared with chickens with the CC genotype (Tables 3 and 4). Therefore, our findings indicate that there is need to increase the selection intensity for ND resistance in Indonesian local chickens to obtain higher *T* allele frequency. Additionally, chicken breeders worldwide can also consider the TT genotype on position 163 bp of NRAMP1 gene as a marker for ND resistance.

**TABLE 3:** Association of the NRAMP‐1 gene with ND antibody titer and white blood cell parameters.

Genotype	ND antibody titer	Leukocyte (cell/uL)	Heterophil (%)	Eosinophil (%)	Lymphocyte (%)	Monocyte (%)	H/L
CC	50.45614^c^	9878.4314	40.4706^a^	2.6863	50.4314^c^	6.4118	0.8189^a^
TC	275.2^b^	9407.5758	29.6364^b^	3.3939	60.4242^b^	6.5455	0.4984^b^
TT	585.8182^a^	9910.6383	22.2766^c^	2.7660	68.3191^a^	6.6383	0.3284^c^
SEM	34.899	149.19832	0.80966	0.13973	0.78261	0.20883	0.022
*p* value	0.000	0.369	0.000	0.111	0.000	0.896	0.000

*Note:* Means with different superscripts differ significantly at the level *p* < 0.05.

**TABLE 4:** The effect of the additive variance of the NRAMP‐1 locus on ND antibody titer and leukogram parameters in local chickens.

Genotype	ND antibody titer	Heterophil (%)	Lymphocyte (%)	H/L
CC	50.45614	40.4706	50.4314	0.8189
CT	275.2	29.6364	6.4242	0.4984
TT	585.8182	22.2766	68.3191	0.3284
Point of origin (O)	16.819	13.4900	16.81	0.2730
Expected mean genotype value (m)	282.329	17.7240	42.5560	0.2890
Observed mean genotype value (M)	299.147	31.2140	59.3660	0.5620
Gene frequency				
C	0.515	0.515	0.515	0.515
T	0.485	0.485	0.485	0.485
Gene effect				
*α*1 (C)	−139.751	4.005	−4.091	0.101
*α*2 (T)	126.619	−5.145	4.885	−0.146

The calculation of the average gene effect showed that the C allele (*α*1) genetically decreases the synthesis of ND antibody titers and reduces the percentage of lymphocytes. The *T* allele (*α*2) increases ND antibody titers and increases the percentage of lymphocytes, thus lowering the H/L ratio, which indicates a reduction in stress levels in chickens. If the *T* allele increases in the population, the population’s genotype mean value will change by (*α*2). In contrast, if the C allele increases, the population’s genotype mean value will change by (*α*1). Based on these results, *T* is the most favorable allele for the selection of ND resistance in chickens.

Table 5 shows the breeding values and genetic variance affecting ND antibody titers and leukogram parameters in local chickens. The effects of genes cannot be measured individually, so the breeding value is always expressed as the sum of the average effects of all genes that influence the observed trait (Pirchner, 1981).

**TABLE 5:** Breeding values and the additive and dominant genetic variance effects based on the NRAMP‐1 locus on ND antibody titers and leukogram parameters in local chickens.

Breeding value	ND antibody titer	Heterophil (%)	Lymphocyte (%)	H/L
CC	−279.50112	8.010	−8.1823	0.2027
CT	−13.1312	−1.1397	0.7936	−0.0448
TT	253.2387	−10.2898	9.7695	−0.2924
Additive variance				
CC	30413.4870	24.9801	26.0647	0.0160
CT	43.4364	0.3272	0.1586	0.0005
TT	23008.4211	37.9874	34.2430	0.0307
Dominance variance				
CC	369.5546	0.6049	0.2206	0.0011
CT	29.4710	0.0482	0,017589915	0.0001
TT	401.0061	0.6564	0,239342665	0.0012
Genetic variance				
CC	30783.0416	25.5851	26.2853	0.0171
CT	72.9074	0.3755	0.1762	0.0006
TT	23409.4272	38.6439	34.4823	0.0319
Total additive variance	53465.3445	63.2948	60.4663	0.0472
Total dominance variance	800.0317	1.3096	0.4775	0.0024
Total genetic variance	54265.3762	64.6045	60.9439	0.0496

The breeding values for ND antibody titer showed that chickens with the homozygous TT genotype have higher breeding values compared to other genotypes, as well as for the percentage of lymphocytes trait. The obtained breeding values indicate that the homozygous TT genotype at the NRAMP1 locus has a higher genetic potential to be inherited by its offspring. Therefore, the selection for ND resistance using the favorable genotype, TT, will result in a rapid genetic improvement since the genotype has a high breeding value. Furthermore, our findings revealed that the genetic variance of ND titer trait is higher than for the percentage of heterophils, lymphocytes, and H/L ratio based on the NRAMP1 locus. These findings indicate high genetic diversity in the chickens, which is important for adaptation and survival under various environmental conditions.

## 4. Conclusion

This study revealed that the NRAMP1 can be considered a promising candidate gene for improving ND resistance in poultry breeding programs through MAS . This study recommends further studies to investigate chickens that are challenged with a virulent ND strain to validate findings from this research. Furthermore, future studies should consider sequencing the complete fragment of the NRAMP1 gene.

## Funding

This study was funded by Jenderal Soedirman University, no. 26.759/UN23.35.5/PT.01/II/2024.

## Conflicts of Interest

The authors declare no conflicts of interest.

## Data Availability

The data that support the findings of this study are available from the corresponding author upon reasonable request.
